# Nothing can replace polyunsaturated lipids

**DOI:** 10.7554/eLife.108249

**Published:** 2025-08-11

**Authors:** Sofia Rodriguez-Gallardo, Takeshi Harayama

**Affiliations:** 1 Department of Cell Biology, Faculty of Biology, University of Seville Seville Spain; 2 Instituto de Biomedicina de Sevilla (IBiS), Hospital Universitario Virgen del Rocío/CSIC/Universidad de Sevilla Seville Spain; 3 Institut de Pharmacologie Moléculaire et Cellulaire, Université Côte d’Azur, CNRS, Inserm Valbonne France

**Keywords:** desaturase, polyunsaturated fatty acids, forward genetics, HIF-1, EGL-9, *C. elegans*

## Abstract

Genetic studies reveal that polyunsaturated lipids do more than simply increase the fluidity of the cell membrane.

**Related research article** Kaper D, Radović U, Bergh PO, Qvist A, Henricsson M, Borén J, Pilon M. 2025. Forward genetics in *C. elegans* reveals genetic adaptations to polyunsaturated fatty acid deficiency. *eLife* **13**:RP104181. doi: 10.7554/eLife.104181.

Every cell in the human body is surrounded by a semi-permeable membrane that needs to protect the contents of the cell while also allowing essential nutrients and ions to pass through.

Compounds called lipids are important components of these membranes, and the saturation levels of these lipids influence the fluidity of the membrane – that is, the sufficient movement of molecules within it. For example, saturated lipids do not have double bonds in their fatty acid tails, so they make membranes less fluid (molecules move more slowly) than monounsaturated lipids (which have one double bond) and polyunsaturated lipids (which have two or more double bonds).

Polyunsaturated lipids have essential roles in health and disease, but it remains unclear how exactly multiple double bonds affect the function of a cell (Lee-Okada et al., 2025). The lipids in the cell membrane form a bilayer, and experiments in cells have shown that monounsaturated lipids alone can create such a structure. Moreover, in many organisms, a single double bond in the lipids is sufficient to make membranes fluid enough to ensure the survival (Harayama and Antonny, 2023). Other experiments on a synthetic minimal organism derived from a bacterium have shown that it can survive if it contains just two types of lipids, neither of which has to be a polyunsaturated lipid (Justice et al., 2024). If polyunsaturated lipids are not required for the formation of bilayers, or for making membranes fluid, why are they so important?

Studying the fundamental roles of polyunsaturated lipids in mammalian cells presents several challenges. While it is possible to disrupt the production of polyunsaturated lipids, cells inevitably obtain them from the serum found in cell culture media. Similar challenges exist for studies in live organisms (Lee-Okada et al., 2025).

The roundworm *Caenorhabditis elegans* is an excellent model that overcomes these hurdles. In contrast to mammals, *C. elegans* can produce new polyunsaturated lipids by converting monounsaturated lipids into polyunsaturated ones with the help of an enzyme called FAT-2 (Watts and Browse, 2002). By genetically modifying the gene for FAT-2, it is thus possible to manipulate the ability of the worms to generate polyunsaturated lipids, and to study the consequences. Additionally, these lab worms consume *Escherichia coli* as their food, which does not provide them with polyunsaturated lipids. Now, in eLife, Marc Pilon of the University of Gothenburg and colleagues – including Delaney Kaper as first author – report on a thorough genetic study for polyunsaturated lipid functions in *C. elegans* (Kaper et al., 2025).

Since the complete loss of FAT-2 in *C. elegans* is lethal, they used a mutant with partial FAT-2 activity that produces less than 10% of normal polyunsaturated lipid levels. The mutants grew poorly and had decreased membrane fluidity but, critically, remained viable. This enabled the researchers to test how their phenotypes can be rescued, thus revealing polyunsaturated lipid functions. Kaper et al. found that mutants lacking FAT-2 did not resume growing even after membrane fluidity was restored. This indicates that polyunsaturated lipids play important roles that extend beyond improving membrane fluidity.

Kaper et al. then used a method called ‘genetic suppressor screening’ to investigate suppressor mutations that can lessen or even reverse an existing mutant phenotype. They identified two classes of mutations that alleviated the growth defects in *fat-2* mutants. The first group included mutations within the *fat-2* gene, which partially restored the activity of the FAT-2 enzyme. The second group involved mutations in the HIF-1 pathway (a key mechanism that enables organisms to low oxygen conditions), which led to an increase in ferrous iron.

Since iron is a cofactor for FAT-2, these mutations rescued phenotypes by boosting FAT-2 activity. Together, the findings show that the only mutations capable of compensating for FAT-2 deficiency are those that restore FAT-2 function and, consequently, polyunsaturated lipid synthesis (Figure 1). In other words, it was impossible to rescue the mutants without restoring lipid composition.

**Figure 1.**
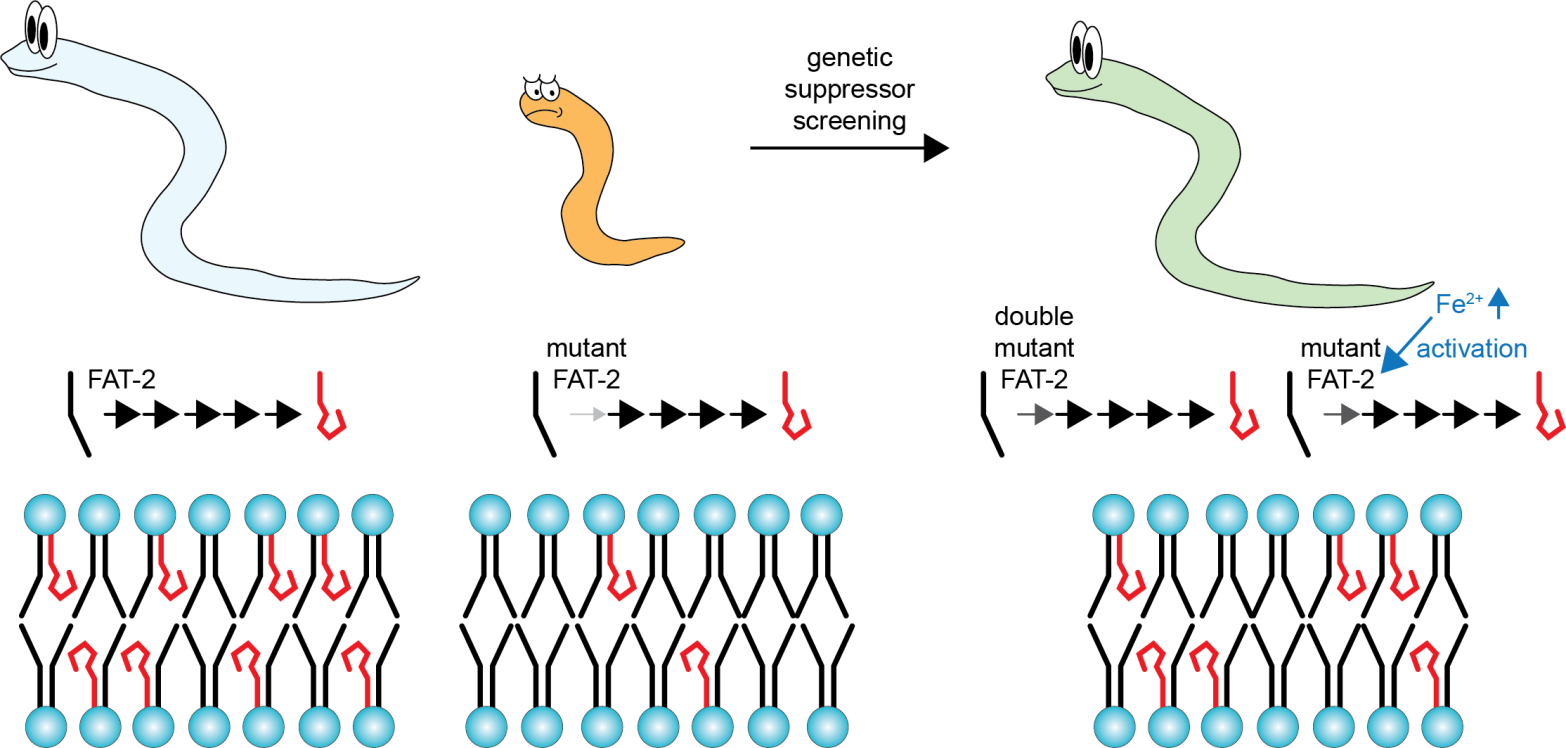
Exploring the role of polyunsaturated lipids. Lipids in the cell membrane form bilayer structures in which the hydrophilic heads (blue circles) of the lipids are on the outside, and the hydrophobic tails (red hooks for polyunsaturated tails; black lines for monounsaturated or saturated tails) are on the inside. Left: wild-type *C. elegans* are rich in polyunsaturated lipids thanks to FAT-2 enzyme, which increases the fluidity of the lipid bilayer and the cell membrane. Middle: mutant worms lacking the *fat-2* gene have significantly lower levels of polyunsaturated lipids due to weak FAT-2 activity, which reduces fluidity. Right: using a method called ‘genetic suppressor screening’, Kaper et al. found that *fat-2* mutants can be restored by two types of mutations, which lead to an increase in the number of polyunsaturated lipids. Mutations in the *fat-2* gene can restore the activity of FAT-2, while mutations in another pathway lead to increased levels of iron (Fe^2+^), which acts as a cofactor to the mutated FAT-2.

The study by Kaper et al. offers some important insights. First, it demonstrates that polyunsaturated lipids do more than just help keep cell membranes fluid. Second, the lack of polyunsaturated lipids appears to impact many biological processes. Since the suppressor screening did not identify any mutations capable of rescuing the phenotypes without restoring the lipid composition, it seems likely that polyunsaturated lipids have a wide range of vital functions.

While Kaper et al. highlight the importance of polyunsaturated lipids, it remains unclear how these fats regulate growth in *C. elegans*. Moreover, the extremely slow growth of the *fat-2* mutants may have biased the genetic suppressor screening towards detecting mutations that directly restore lipid composition. In the future, using *fat-2* mutants with milder defects could make it possible to identify other genetic pathways that are not directly implicated in the restoration of lipid composition, which may reveal previously unknown biological functions of polyunsaturated lipids.

Although genome-wide screenings are now common in mammalian systems, they typically only detect the effects of gene loss or overexpression (Roberts et al., 2023). As a result, identifying gain-of-function point mutations that rescue phenotypes remains a challenge. Nevertheless, the study by Kaper et al. leverages powerful genetic tools available in *C. elegans* to explore the role of polyunsaturated lipids and identify suppressor mutants. This approach paves the way for discovering fundamental insights into how the double bonds in polyunsaturated lipids can be linked to biological functions beyond simply maintaining membrane fluidity.
